# The Lantibiotic Peptide Labyrinthopeptin A1 Demonstrates Broad Anti-HIV and Anti-HSV Activity with Potential for Microbicidal Applications

**DOI:** 10.1371/journal.pone.0064010

**Published:** 2013-05-28

**Authors:** Geoffrey Férir, Mariya I. Petrova, Graciela Andrei, Dana Huskens, Bart Hoorelbeke, Robert Snoeck, Jos Vanderleyden, Jan Balzarini, Stefan Bartoschek, Mark Brönstrup, Roderich D. Süssmuth, Dominique Schols

**Affiliations:** 1 Rega Institute for Medical Research, University of Leuven, Leuven, Belgium; 2 Centre of Microbial and Plant Genetics, University of Leuven, Leuven, Belgium; 3 Department of Bioscience Engineering, Antwerp University, Antwerp, Belgium; 4 Sanofi R&D, Frankfurt am Main, Germany; 5 Technische Universität Berlin, Fakultät II – Institut für Chemie; Berlin, Germany; Ghent University, Belgium

## Abstract

Lantibiotics are peptides, produced by bacteria, that contain the noncanonical amino acid lanthionine and many of them exhibit antibacterial activities. The labyrinthopeptin A1 (LabyA1) is a prototype peptide of a novel class of carbacyclic lantibiotics. Here, we extensively evaluated its broad-spectrum activity against HIV and HSV *in vitro,* studied its mechanism of action and evaluated potential microbicidal applications. LabyA1 exhibited a consistent and broad anti-HIV activity (EC_50_s: 0.70–3.3 µM) and anti-HSV activity (EC_50_s: 0.29–2.8 µM) in cell cultures. LabyA1 also inhibited viral cell-cell transmission between persistently HIV-infected T cells and uninfected CD4^+^ T cells (EC_50_∶2.5 µM) and inhibited the transmission of HIV captured by DC-SIGN^+^-cells to uninfected CD4^+^ T cells (EC_50_∶4.1 µM). Time-of-drug addition studies revealed that LabyA1 acts as an entry inhibitor against HIV and HSV. Cellular and virus binding studies combined with SPR/FLIPR technology showed that LabyA1 interacted with the HIV envelope protein gp120, but not with the HIV cellular receptors. LabyA1 also demonstrated additive to synergistic effects in its anti-HIV-1 and anti-HSV-2 activity with anti(retro)viral drugs in dual combinations such as tenofovir, acyclovir, saquinavir, raltegravir and enfuvirtide. LabyA1 can be considered as a novel lead peptide as it had profound antiviral activity against HIV and HSV. Pre-treatment of PBMCs with LabyA1 neither increased the expression of the activation markers CD69 and CD25, nor enhanced HIV replication, nor significantly induced various inflammatory cytokines/chemokines. LabyA1 also did not affect the growth of vaginal *Lactobacilli* populations. Based on the lack of toxicity on the vaginal *Lactobacillus* strains and its synergistic/additive profile in combination with clinically approved anti(retro)virals, it deserves further attention as a potential microbicide candidate in the prevention of sexual transmitted diseases.

## Introduction

At present, 34 million people are estimated to live with HIV (human immunodeficiency virus) and approximately 2.5 million novel infections occurred worldwide in 2011 [1]. To impede HIV transmission and infection, condom use, male circumcision and behavioral interventions are available methods, but novel pre-exposure prevention (PrEP) strategies are needed such as vaginal/rectal gels, creams, pills and intravaginal ring systems [2].

The first break-through in the field of microbicidal research was the outcome of the CAPRISA 004 (Centre for the AIDS Program of Research In South Africa) trial, using a 1% vaginal tenofovir gel which reduced the transmission of HIV by 39% and of herpes simplex virus type 2 (HSV-2) by 51% [3]. However, the VOICE (Vaginal and Oral Interventions to Control the Epidemic) study halted the oral tenofovir and tenofovir gel arms, because interim data analysis showed that the results were not so promising [4]. The focus on PrEP is mainly based on reverse transcriptase inhibitors (RTIs; e.g. tenofovir, dapivirine) [3], [5], [6]. Compared to RTIs, entry inhibitors (EIs) have the benefit that they target HIV in the lumen of the vagina before genital tissue penetration and dissemination towards the lymph nodes. The probability of HIV-1 transmission per coital act is very low (0.0001–0.004) and depends on the route of transmission (male/male; male/female or female/male), however animal models have shown that infection is established relatively quickly (within an hour) at the mucosal surface [7]. An increase in the transmission rate could be observed with disruption of the (vaginal) epithelial integrity by e.g. ulceration, bacterial vaginosis and hormonal status [7], [8].

HIV infection starts with the attachment of the trimeric envelope glycoprotein gp120 to three CD4 receptor molecules. This leads to conformational changes inside gp120 and subsequent interactions with the chemokine receptors CXCR4 and/or CCR5 will take place. After these coreceptor binding events, membrane fusion is further induced by gp41 [9].

HSV-2 infection causes genital ulcers and appears to act synergistically with HIV. It has been shown that genital lesions and altered innate mucosal immunity caused by HSV-2 are important cofactors to increase the rate of HIV transmission and infection [10], [11]. Therefore, a product that inhibits HIV and HSV would have potential benefits in the prophylaxis against these sexually transmitted viruses. As for HIV, HSV entry is also a multistep process, whereby the HSV virions first attach with their glycoprotein B (gB) and/or gC to the heparan sulfate proteoglycans followed by the interaction of gD with a gD receptor. This results in conformational changes inside gD and triggers (receptor-induced triggering) the activation of the heterodimer gH/gL to bind and activate the fusion activity of the gB envelope protein [12], [13].

**Figure 1:**
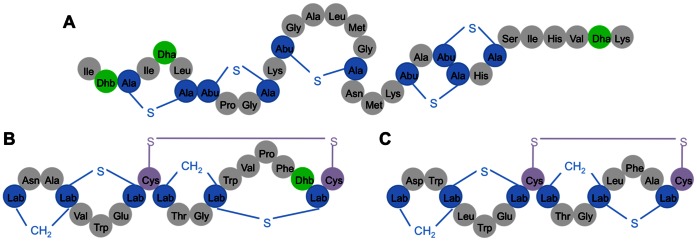
Primary structures of the lantibiotics used in this study. Primary structures of the lantibiotic peptides (A) nisin, (B) labyrinthopeptin A1 (LabyA1) and (C) Labyrinthopeptin A2 (LabyA2). Lab, Dhb and Dha are the abbreviations of the unusual amino acids labionin, didehydrobutyrine and didehydroalanine. Abu-S-Ala in nisin is methyllanthionine.

Lantibiotics are ribosomally synthesized peptides, produced by *Staphylococci*, *Lactobacillus* and *Actinomycetes.* Posttranslational modifications generate the amino acids lanthionine or methyllanthionine, that are characterisitic for lantibiotics [14]. The most studied lantibiotic nisin (belonging to the type I lantibiotics, Fig. 1A) is widely used as a food preservative (European food additive number E234) for more than 40 years [15]. The labyrinthopeptins are a novel class of carbacylic type III lantibiotics containing labionin (Lab, Fig. 1B–C), a posttranslationally modified triamino acid [16]. In a first set of studies, pronounced activity in a neuropathic pain mouse model and moderate anti-herpetic activity was reported for labyrinthopeptin A2 (LabyA2; MW = 1922.6 Da; Fig. 1C) [16].

In this study, we focus on the biological properties of labyrinthopeptin A1 (LabyA1; Fig. 1B). LabyA1 (MW = 2073.7 Da) was isolated from the actinomycete *Actinomadura namibiensis* DSM 6313 [17], [18], and its biosynthesis was investigated in subsequent studies [16], [19], [20], [21]. Here, we showed its broad-spectrum anti-HIV and anti-HSV activity as well as its potential for microbicidal applications in the prevention of infection/transmission of the sexually transmitted copathogens HIV and HSV.

## Results

### Broad-spectrum Anti-HIV and Anti-HSV Activity of Labyrinthopeptins

The lantibiotic peptide LabyA1 showed a very consistent anti-HIV activity against various widely-used and cell-line adapted HIV-1 strains such as X4 NL4.3 and R5 BaL with a median EC_50_ of 1.9 µM (range from 0.79 to 3.3 µM; Table 1). The observed antiviral activity is also independent of the viral coreceptor (CXCR4 or CCR5) use (Table 1).

**Table 1:** Anti-HIV activity (EC_50_) of labyrinthopeptin A1 (LabyA1) against laboratory-adapted and drug-resistant HIV strains determined in various cell types.

Cell lines	HIV (Coreceptor use)	EC_50_ (µM ± SEM)
MT-4^a^	HIV-1 NL4.3 (X4)	1.7±0.4
MT-4^a^	HIV-1 IIIB (X4)	2.0±0.6
MT-4^a^	HIV-2 ROD (R5/X4)	1.9±0.7
PBMCs^b^	HIV-2 ROD (R5/X4)	1.0±0.3
PBMCs^b^	HIV-1 HE (R5/X4)	1.8±0.3
PBMCs^b^	HIV-1 NL4.3 (X4)	0.79±0.09
PBMCs^b^	HIV-1 BaL (R5)	3.3±0.4
MDM^b^	HIV-1 BaL (R5)	2.4±0.8
*Median EC_50_*		*1.9 µM*
MT-4^a^	HIV-1 NL4.3^2G12res.^ (X4)^c^	2.2±0.3
MT-4^a^	HIV-1 NL4.3^AMD3100res.^ (X4)^c^	2.4±0.5
MT-4^a^	HIV-1 NL4.3^T20res.^ (X4)^c^	2.3±0.3
MT-4^a^	HIV-1 NL4.3^AZTres.^ (X4)^c^	1.1±0.1
MT-4^a^	HIV-1 NL4.3^RALres.^ (X4)^c^	0.88±0.08
MT-4^a^	HIV-1 IIIB^FGMres.^ (X4)^c^	1.9±0.1
*Median EC_50_*		*2.1 µM*

a Viral replication measured by MTS/PES method. Mean ± SEM out of 2–6 independent experiments is shown.

b Viral replication measured by p24 HIV-1 or p27 HIV-2 Ag ELISA. Mean ± SEM out of 2–8 independent PBMC donor experiments is shown.

c HIV-1 strains resistant to the anti-carbohydrate mAb 2G12, CXCR4 antagonist AMD3100, gp41 fusion inhibitor enfuvirtide (T20), RTI azidothymidine (AZT), INI raltegravir (RAL) and the CD4/gp120 binding inhibitor feglymycin (FGM).

**Figure 2:**
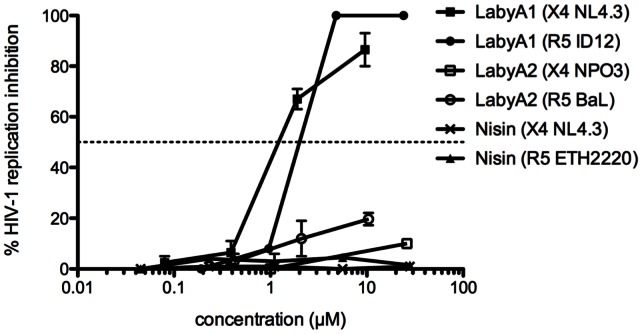
Lack of anti-HIV activity of nisin and LabyA2. Dose-dependent effects of LabyA1, LabyA2 and nisin against HIV-1 X4 NL4.3 replication in MT-4 cells. HIV-1 X4 strain NPO3 and R5 strains BaL, ID12 and ETH2220 replication is evaluated in PBMCs. Data represent mean ± SEM for up to 3 independent experiments.

As the envelope protein gp120 of HIV-1 is characterized by an enormous heterogeneity we therefore evaluated the antiviral activity of LabyA1 against 9 different HIV-1 clinical isolates (8 from group M and 1 from group O). LabyA1 showed again a very consistent anti-HIV-1 activity with a median EC_50_ of 1.0 µM (range from 0.70 to 1.9 µM; Table 2). In contrast, the EC_50_s of LabyA2 and the lantibiotic nisin (here used as reference lantibiotic), against HIV-1 were, respectively, >26 µM (>50 µg/ml LabyA2) and >29 µM (>100 µg/ml nisin) (Fig. 2).

**Table 2:** Broad-spectrum anti-HIV-1 (EC_50_) activity of LabyA1 against various HIV-1 clinical isolates representing different subtypes evaluated in PBMCs.

Group	HIV-1 (coreceptor use)	Subtype	EC_50_ (µM)^a^
M	UG273 (clinical isolate; R5)	A	1.9±0.1
	US2 (clinical isolate; R5/X4)	B	0.70±0.19
	CI#17 (clinical isolate; X4)	B	1.0±0.2
	DJ259 (clinical isolate; R5)	C	0.94±0.43
	ETH2220 (clinical isolate; R5)	C	0.78±0.06
	UG270 (clinical isolate; X4)	D	1.5±0.1
	NPO3 (clinical isolate; X4)	E	1.6±0.3
	ID12 (clinical isolate; R5)	A/E	1.2±0.8
O	BCF-06 (clinical isolate; X4)		0.83±0.33^b^
*Median EC_50_*			*1.0 µM*

a EC_50_s measured by p24 Ag ELISA. Mean ± SEM out of 2–5 independent PBMC donor experiments is shown.

b EC_50_ measured by p27 Ag ELISA. Mean ± SEM out of 4 independent PBMC donor experiments is shown.

Next, we investigated the activity of LabyA1 against various HSV strains. As shown in Table 3, LabyA1 also showed a consistent anti-HSV activity since it inhibited the viral induced cytopathic effect (CPE) in the human embryonic lung (HEL) fibroblast cell cultures with consistent EC_50_s ranging between 0.29 and 2.8 µM. Cidofovir and acyclovir were always included as reference compounds (Table 3). As previously observed, also LabyA2 inhibited HSV-1 and HSV-2 replication [16], but as shown in Tables 3 and 4, LabyA2 was on average at least 10-fold less potent than LabyA1 and nisin displayed no antiviral activity. The anti-herpes virus activity of LabyA1 was thus comparable with the anti-herpetic drugs acyclovir and cidofovir, with no marked differences in the inhibition between the two herpes viruses HSV-1 and HSV-2.

**Table 3:** Anti-HSV type-1 and HSV type-2 activity (EC_50_) of LabyA1.

Virus strain		EC_50_ (µM)^a^		
		LabyA1	Cidofovir	Acyclovir
HSV-1 wild-type	KOS (reference strain)	0.56±0.05	0.48±0.11	0.18±0.09
	RV-174 (clinical isolate)	2.7±1.0	1.3±0.1	0.31±0.02
	RV-175 (clinical isolate)	2.8±0.5	1.1±0.1	0.18±0.03
	C559142 (clinical isolate)	0.45±0.02	0.53±0.09	0.09±0.02
*Median EC_50_*		*1.6 µM*	*0.82 µM*	*0.18 µM*
HSV-1 TK^−b^	RV-294 (clinical isolate)	0.31±0.00	0.30±0.02	25±0.0
	RV-179 (clinical isolate)	1.9±0.3	0.39±0.12	89±0.0
	RV-117 (clinical isolate)	1.9±0.6	0.50±0.12	>79
*Median EC_50_*		*1.9 µM*	*0.39 µM*	*>79 µM*
HSV-2 wild-type	G (reference strain)	0.32±0.05	0.60±0.10	0.27±0.01
	RV-124 (clinical isolate)	1.4±0.4	1.3±0.3	0.40±0.06
	RV-24 (clinical isolate)	0.41±0.28	1.1±0.3	0.13±0.09
*Median EC_50_*		*0.41 µM*	*1.1 µM*	*0.27 µM*
HSV-2 TK^−b^	RV-129 (clinical isolate)	0.32±0.19	0.55±0.20	74±12
	BA19026589 (clinical isolate)	0.29±0.10	0.95±0.24	29±9
*Median EC_50_*		*0.31 µM*	*0.75 µM*	*52 µM*

a EC_50_, 50% effective concentration or compound concentration required to reduce virus-induced cytopathicity (CPE) by 50%. Mean EC_50_± SEM up to 3 independent experiments are shown. Cidofovir and acyclovir are used as reference compound.

b TK^−^: thymidine kinase-deficient HSV strains (resistant to acyclovir, see [32] for more detail).

**Table 4:** Anti-HSV type 1 and HSV type 2 activity (EC_50_) of LabyA2, nisin and acyclovir.

Virus strain^c^		EC_50_ (µM)^a^		
		LabyA2	Nisin	Acyclovir
HSV-1 wild-type	KOS	9.2±2.0	>15	0.18±0.09
	RV-174	17.5±2.4	>15	0.31±0.02
	RV-175	15.6±2.4	>15	0.18±0.03
	C559142	4.4±0.4	>15	0.09±0.02
*Median EC_50_*		*12.4 µM*	*>15 µM*	*0.18 µM*
HSV-1 TK^−b^	RV-294	3.7±0.2	>15	25±0.0
	RV-179	11.0±0.6	>15	89±0.0
	RV-117	10.7±3.2	>15	>79
*Median EC_50_*		*10.7 µM*	*>15 µM*	*>79 µM*
HSV-2 wild-type	G	1.9±0.2	>15	0.27±0.01
	RV-124	6.7±1.5	>15	0.40±0.06
	RV-24	5.5±2.0	>15	0.13±0.09
*Median EC_50_*		*5.5 µM*	*>15 µM*	*0.27 µM*
HSV-2 TK^−b^	RV-129	3.7±1.6	>15	74±12
	BA19026589	2.7±0.3	>15	29±9
*Median EC_50_*		*3.2 µM*	*>15 µM*	*52 µM*

a EC_50_, 50% effective concentration or compound concentration required to reduce virus-induced cytopathicity (CPE) by 50%. Mean EC_50_± SEM up to 3 independent experiments are shown. Acyclovir is used as reference compound.

b TK^−^: thymidine kinase-deficient HSV strains (resistant to acyclovir, see [32] for more detail).

c For more detail about the viral strains, see Table 3.

In addition, none of the tested lantibiotics showed antiviral activity against the influenza viruses H_1_N_1_, H_3_N_2_ and influenza B (EC_50_, >20 µM).

### LabyA1 Inhibits HIV-induced Cell-cell Syncytia Formation

**Figure 3:**
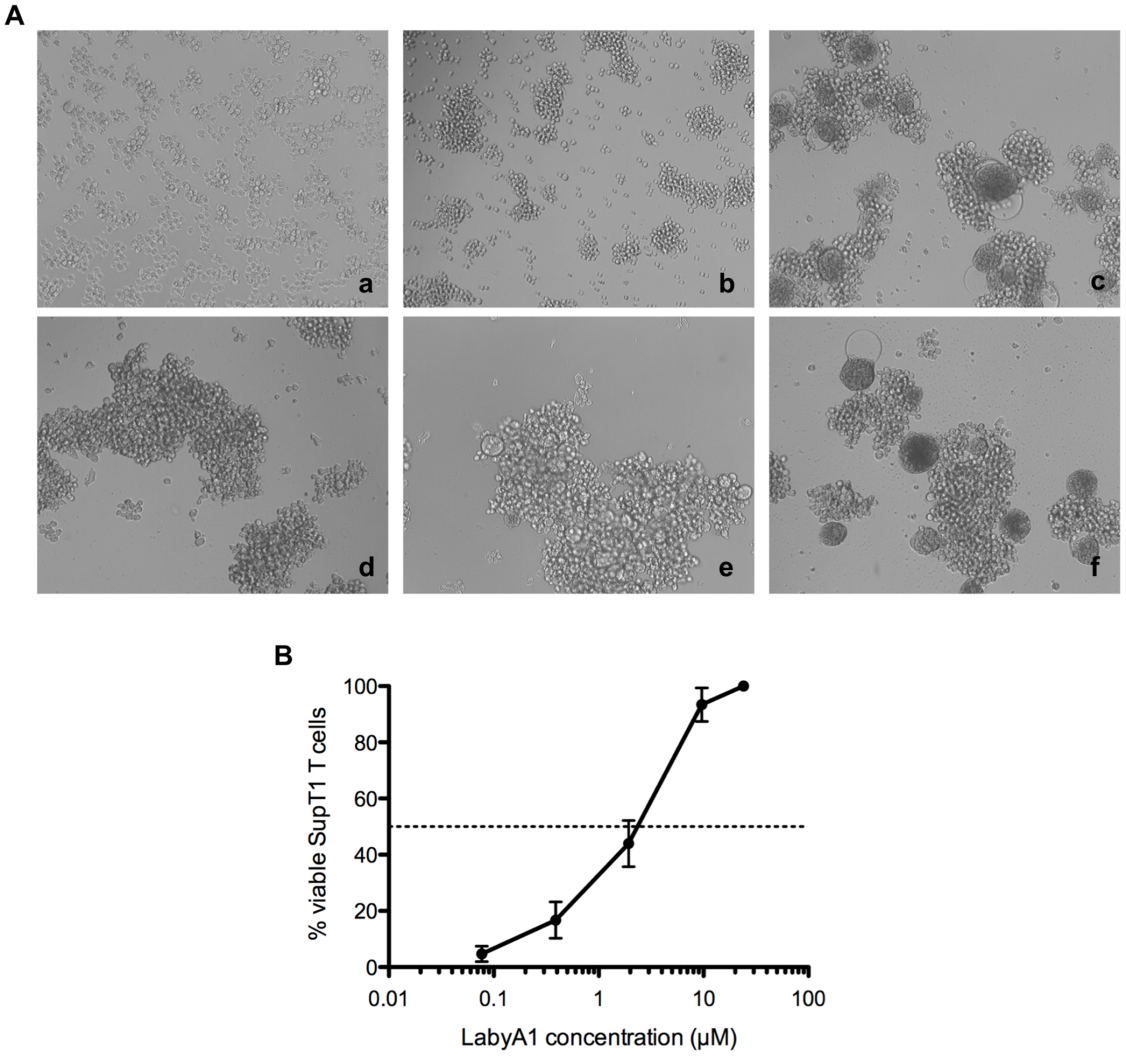
Inhibitory activity of LabyA1 against the cell-to-cell HIV transmission. (A) Light microscopical pictures of (a) persistently HIV-infected HUT-78/IIIB cells, (b) non-infected CD4^+^ target T cells, (c) giant cell formations after coculture of HUT-78/IIIB and SupT1 cells, (d) coculture of HUT-78/IIIB and SupT1 cells in the presence of 24 µM LabyA1, (e) 4.8 µM LabyA1 and (f) 0.96 µM LabyA1. One representative experiment is shown. Magnification x10/0.25. (B) Percentage of living SupT1 T cells after 24 h of coculture with HUT-78/IIIB cells in the presence of LabyA1. To discriminate SupT1 T cells from HUT-78/IIIB cells, the expression of the CD28 marker was measured by flow cytometry. The mean ± SEM out of 4 independent experiments is shown.

During HIV transmission, CD4^+^ T cells cannot only be infected by cell-free virions but, importantly, also by cell-cell contacts with donor HIV-infected T cells. Mixing persistently HIV-infected cells (HUT-78/IIIB cells) with non-infected CD4^+^ target T cells (SupT1 cells), enormous syncytia or giant cells are formed in less than 20 h, as shown by light microscopical pictures in Fig. 3A (panels a–c). At a concentration of 24 µM of LabyA1, giant cell formation was completely inhibited (Fig. 3A panel d). At 4.8 µM, some giant cells were formed (Fig. 3A, panel e), however, the number and size of these giant cells were less as compared to the positive control. At a 5-fold lower concentration of LabyA1 (0.96 µM), no activity was observed anymore in this cell-cell fusion assay (Fig. 3A, panel f). In addition, we quantified the number of viable SupT1 cells after cocultivation with HUT-78/IIIB cells in the presence of LabyA1. We could distinguish flow cytometrically SupT1 cells (CD28^+^) from HUT-78/IIIB cells (CD28^−^) by staining the cell cocultures with an anti-CD28 mAb. In the presence of LabyA1, the percentage of SupT1 T cells that survived increased dose-dependently and an EC_50_ of 2.5±0.6 µM was calculated (Fig. 3B).

### Inhibitory Effects of LabyA1 on the Entry of HIV and HSV

**Figure 4:**
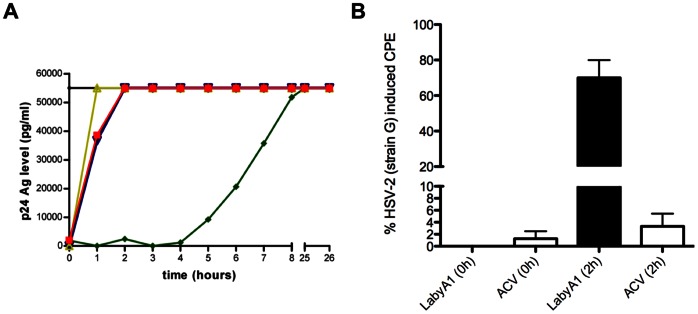
LabyA1 interferes with the viral replication at an early time point. (A) MT-4 cells were infected with HIV-1 IIIB and test compounds were added at different time points post-infection. After 31 h, HIV-1 IIIB replication was measured by p24 Ag ELISA. The colored lines represent the positive control in black, 48 µM LabyA1 in red, 421 µM dextran sulfate 8000 in yellow, 6 µM AMD3100 in blue and 7.5 µM nevirapine in green. (B) HEL cells were infected with HSV-2 strain G and either 19 µM of LabyA1 or 35 µM of ACV was added respectively at 0 h and 2 h post-infection. After 3 days, HSV-2 induced CPE was scored microscopically. Data represent mean values ± range of 2 experiments, each performed in duplicate.

A time-of-drug addition (TOA) experiment was performed to determine the antiviral target of LabyA1. From the polyanionic compound dextran sulfate 8000 (DS, MW 8000), it is known that it can only inhibit HIV replication at the time of infection. The antiviral activity was completely lost if added 1 h after infection (Fig. 4A). Addition of the CXCR4 antagonist, AMD3100, 2 h post-infection resulted in complete loss of antiviral activity, while the non-nucleoside reverse transcriptase inhibitor (NNRTI) nevirapine kept its full activity when administered up to 4 h post-infection. As seen in Fig. 4A, LabyA1 prevented HIV infection at an early time point somewhat comparable with AMD3100. These results indicate that LabyA1 interferes with the HIV entry process, presumably by acting as an adsorption/coreceptor/fusion inhibitor.

Additionally, we determined the antiviral activity of LabyA1 against 6 different drug-resistant HIV strains (4 EIs: the anti-gp120 mAb 2G12, the CXCR4 inhibitor AMD3100, the gp41 inhibitor T20 and the CD4/gp120 inhibiting bacterial peptide feglymycin, 1 RTI: azidothymidine (AZT) and 1 INI: raltegravir). As shown in Table 1, no loss in anti-HIV activity was observed against these viruses (EC_50_s ranged between 0.88 and 2.4 µM), compared to their corresponding wild type HIV-1 strains IIIB and NL4.3.

TOA experiments were also performed with the HSV-2 strain G. When high concentrations of our test agent LabyA1 (19 µM) or the DNA polymerase targeting agent acyclovir (ACV; 35 µM) were given simultaneously with the HSV-2 strain G, no CPE or viral replication were observed after 3 days (Fig. 4B). However, addition of the agents after a 2 h adsorption period resulted in a significant decrease (*p* = 0.006) in the antiviral activity of LabyA1, while ACV was still active (*p*>0.05). These HSV experiments clearly indicate that, as for HIV-1, LabyA1 interferes with the viral entry process.

### Lack of Interaction between LabyA1 and the Cellular Receptors CD4, CXCR4 and CCR5

**Figure 5:**
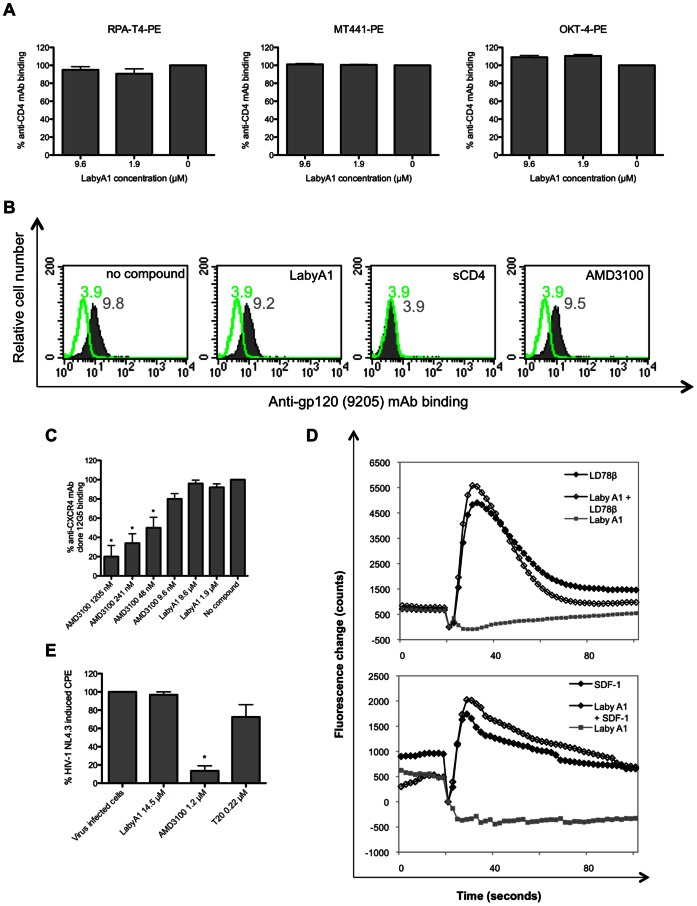
No interaction of LabyA1 with the cellular receptors involved in HIV pathogenesis. (A) CD4^+^ SupT1 cells were incubated with 3 different anti-CD4 mAbs (RPA-T4, MT441 and OKT-4), in the presence of 9.6 µM or 1.9 µM of LabyA1 and antibody binding was measured by flow cytometry. Data represent the mean-values ± SEM of the percentage of anti-CD4 mAb binding of 2–3 independent experiments. (B) Binding of HIV-1 NL4.3, measured by the anti-gp120 (9205) mAb, to CD4^+^ SupT1 cells in the presence of LabyA1, sCD4 and AMD3100. MFI-values of the background fluorescence and virus binding are shown in green and grey histograms, respectively. One representative experiment out of 3 is shown. (C) Interaction with the CXCR4 receptor was measured by flow cytometry after incubation of SupT1 T cells with the anti-CXCR4mAb (12G5) in the presence of 9.6 µM and 1.9 µM of LabyA1. AMD3100 was included as reference compound. Data represent mean-values ± SEM (*n* = 3) of the percentage of CXCR4 mAb binding according to the untreated control. *p<0.05, according to unpaired T-test. (D) Intracellular calcium mobilization in U87.CD4.CCR5 cells (upper panel) and in U87.CD4.CXCR4 cells (lower panel). Fluo-3 loaded cells pre-incubated in the absence or presence of 9.6 µM LabyA1 were stimulated with 10 ng/ml LD78β (upper panel) or 100 ng/ml SDF-1α (lower panel) or with 9.6 µM LabyA1 and the fluorescence change was monitored. One representative experiment out of 3 is shown. (E) MT-4 cells were pre-incubated with LabyA1, AMD3100 or T20, extensively washed and infected with HIV-1 NL4.3. Viral replication was measured 5 days post-infection. Mean CPE values ± SEM up to 5 experiments are shown. Statistical *p<0.05, according to unpaired T-test.

First, investigated whether the main HIV cellular receptor, CD4, is a possible target for LabyA1. We checked if LabyA1 could inhibit the binding of 3 anti-CD4 mAbs on SupT1 T cells: the anti-CD4 mAbs RPA-T4, MT441 and OKT4 that recognize domain 1, 2 and 4, respectively. However, various concentrations of LabyA1 had no effect on the binding of these anti-CD4 mAbs (Fig. 5A), and thus presumably indicating no significant interactions with the CD4 receptor.

Next, we investigated whether LabyA1 could inhibit HIV-1 binding to CD4^+^ T cells. Bound virus was detected using the 9205 mAb, recognizing the tip of the V3 loop on gp120 [22]. HIV-1 NL4.3 binding on SupT1 T cells was observed by flow cytometry and a mean fluorescence intensity (MFI) of 9.8 was measured (Fig. 5B). Addition of 9.6 µM of LabyA1 had no effect on virus binding (MFI of 9.2), while this process was completely inhibited in the presence of sCD4 (10 µg/ml), as the MFI decreased from 9.8 to 3.9, which equals the value of the background fluorescence. Fig. 5B also shows that the virus binding to CD4^+^ T cells was not compromised in the presence of 12 µM of the CXCR4 antagonist AMD3100 (MFI: 9.5).

As based on the TOA studies and virus binding experiments, it was still not excluded that CXCR4 could be a target receptor for LabyA1. Therefore, we incubated SupT1 cells with various concentrations of LabyA1 or AMD3100 and with the anti-CXCR4 mAb clone 12G5. AMD3100 inhibits significantly (*p*<0.05) the interaction of 12G5 mAb with the CXCR4 receptor with an IC_50_ of 40 nM, as described previously [23]. As shown in Fig. 5C, LabyA1 was unable to inhibit the binding of the anti-CXCR4 mAb 12G5 (*p*>0.05). An additional method to detect the interaction of a compound with a chemokine receptor is by measuring a chemokine-induced intracellular calcium signal. After binding to their receptor (e.g. CXCR4 and CCR5), chemokines trigger an intracellular signal transduction cascade, which results in transient cytosolic calcium mobilization. LabyA1 could not induce by itself calcium signaling in U87.CD4.CCR5 (Fig. 5D, upper panel) or U87.CD4.CXCR4 (Fig. 5D, lower panel) cells. LabyA1 could also not inhibit the intracellular calcium flux induced by the chemokines LD78β (CCL3L1) and SDF-1α (CXCL12) in U87.CD4.CCR5 and U87.CD4.CXCR4 cells, respectively (Fig. 5D). These data demonstrate that LabyA1 has no measurable effect on the HIV cellular receptors CD4, CXCR4 and CCR5.

To investigate if LabyA1 interacts in an “aspecific” manner with the cell membrane, we pre-incubated CD4^+^ MT-4 cells with either LabyA1, the CXCR4 inhibitor AMD3100 or the gp41-fusion inhibitor T20 for 2 h at 37°C, then removed the compounds from the cell cultures and then, the cells were infected with HIV-1 NL4.3. After 5 days of culture, the HIV-infected cells were completely destroyed by the virus resulting in 100% CPE. As shown in Fig. 5E, LabyA1 was not able to inhibit viral infection (96.8±3.2% CPE; *p* = 0.374). A comparable observation was made for the gp41 fusion inhibitor T20 (72.6±13.4% CPE; *p* = 0.111). AMD3100 significantly protected the cells, as it interacted with the CXCR4 receptors of the target T cells, and the observed percentage CPE of the AMD3100-pretreated cell culture was 13.5±5.5% CPE (*p* = 0.04) (Fig. 5E). Comparable results were observed using the TZM-bl cell line and HIV-1 NL4.3 (data not shown). Thus, where the compounds were washed away before HIV infection, LabyA1, as T20, did not protect the cells anymore and this suggests strongly that it interacts with the virus and not with the CD4^+^ T cells.

### Interaction of LabyA1 with the Envelope Protein gp120 of HIV

A quantitative way to investigate whether agents bind to viral envelope glycoproteins is the use of surface plasmon resonance (SPR) technology [24]. Binding properties of LabyA1 and nisin were evaluated towards the X4 HIV-1 IIIB, R5 HIV-1 ADA and YU2 gp120. As shown in Table 5, LabyA1 binds with an affinity constant in the lower µM range to X4 and R5 gp120, while nisin did not show a binding signal when exposed to gp120.

**Table 5:** Kinetic data for the interaction of LabyA1 and nisin with immobilized X4 and R5 HIV-1 gp120 envelope proteins.

	KD (µM)	Kon (1/M.s)	Koff (1/s)
LabyA1			
X4 HIV-1 gp120 IIIB	7.8±2.1	(9.6±1.6)E+02	(7.3±0.8)E-03
R5 HIV-1 gp120 ADA	12.3±2.2	(7.2±1.0)E+02	(8.7±1.3)E-03
R5 HIV-1 gp120 YU2	9.8±1.6	(7.3±0.5)E+02	(7.2±0.7)E-03
Nisin			
X4 HIV-1 gp120 IIIB	No binding observed at 31.3 µM		
R5 HIV-1 gp120 ADA	No binding observed at 31.3 µM		
R5 HIV-1 gp120 YU2	No binding observed at 31.3 µM		

K_D_: affinity constant; K_on_: association rate constant; K_off_: dissociation rate constant.

Mean ± SD of 2 independent experiments are shown.

### Activity of LabyA1 in a DC-SIGN-mediated HIV Transmission Assay

**Figure 6:**
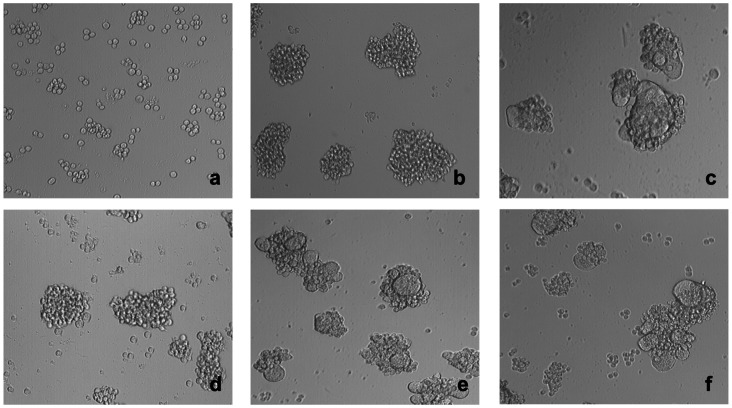
Inhibitory activity of LabyA1 against DC-SIGN-mediated viral transmission. Light microscopical pictures of (a) Raji.DC-SIGN^+^ cells that captured the R5/X4 HIV-1 strain HE (Raji.DC-SIGN/HE), (b) CD4^+^ target C8166 T cells, (c) giant cell formation after 24 h in a coculture of Raji.DC-SIGN/HE cells with C8166 T cells, (d) coculture in the presence of 9.6 µM, (e) 1.9 µM and (f) 0.19 µM of LabyA1. One representative experiment out of 2 is shown. Magnification x10/0.25.

A possible HIV mucosal infection pathway is the transmission of DC-SIGN (dendritic cel-specific ICAM-3 grabbing non-integrin) captured virus to CD4^+^ T cells and we investigated whether LabyA1 could inhibit this pathway. HIV-1 X4/R5 HE was given the opportunity to bind to DC-SIGN on Raji.DC-SIGN^+^ cells (Fig. 6, panel a) and in the meantime CD4^+^ target T cells (C8166, Fig. 6 panel b) were incubated with various concentrations of LabyA1. When HIV-1-captured DC-SIGN^+^ cells were cocultured with the CD4^+^ T cells in the absence of LabyA1, viral transmission could be observed microscopically within 20 h by massive giant cell formation and CD4^+^ T cell destruction (Fig. 6, panel c), and viral replication could be measured (p24 Ag concentration of 263.9 ng/ml). At 9.6 µM, LabyA1 fully protected the cells from giant cell formation (Fig. 6, panel d) and no viral replication was measured (p24 Ag concentration lower than the detection range (<25 pg/ml)), while at 1.9 and 0.19 µM, its inhibitory effect was not detectable (Fig. 6, panels e-f and p24 Ag concentration of 393.7 ng/ml). Based on these data, we can conclude that LabyA1 has a protective effect on the DC-SIGN-mediated transmission and subsequent replication of HIV-1 with a mean EC_50_ of 4.1±0.2 µM.

### Potential Side-effects of LabyA1 on PBMCs

**Figure 7:**
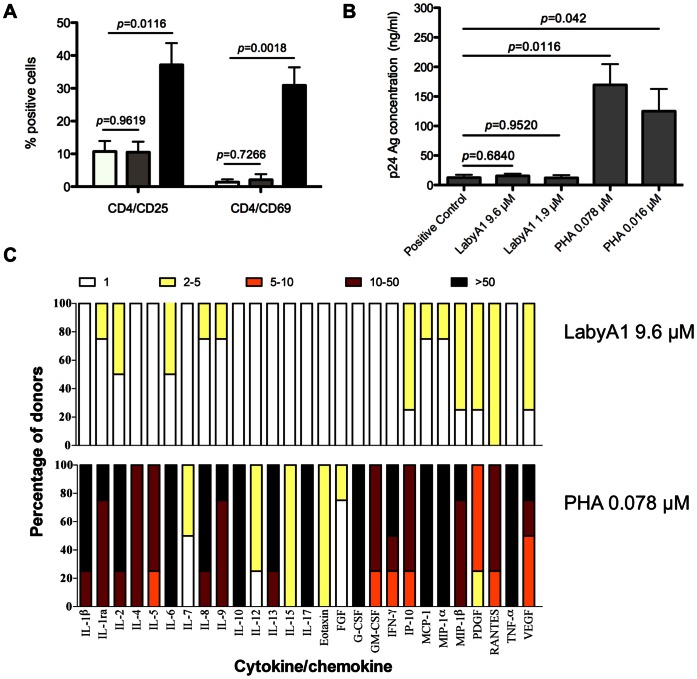
Lack of stimulatory activity of LabyA1 on PBMCs. (A) Freshly isolated PBMCs were incubated with 9.6 µM LabyA1 and 0.016 µM PHA for 3 days and afterwards CD25 and CD69 expression was analyzed using anti-CD25-PE and anti-CD69-PE conjugated mAbs in combination with anti-CD4-conjugated PerCP. Mean ± SEM out of 4 individual donor experiments are shown. The white bars represent untreated conditions, while the grey and black bars indicate, treatment of 9.6 µM of LabyA1 and 0.016 µM of PHA, respectively. *p*-values, according to unpaired T-test. (B) Freshly isolated PBMCs were pre-treated with various concentrations of LabyA1 and PHA for 24 h. After extensive washing, the cells were then infected with HIV-1 R5 BaL in the absence of compounds. The viral replication level at day 7 post-infection was measured using HIV-1 p24 Ag ELISA. Mean ± SEM of 3 independent PBMC donors is shown. *p*-values, according to unpaired T-test (C) Freshly isolated PBMCs from healthy donors were incubated for 24 h with medium only or LabyA1 or PHA. Supernatant was collected and cytokine/chemokine production was determined using the Bio-Plex system. The fold-increase of the cytokine/chemokine concentration in the supernatant of treated PBMCs with respect to the concentrations in the supernatant of untreated PBMCs was determined for 4 individual donors for LabyA1 and PHA. The fold-increase is divided in 5 subgroups: 1-fold increase (white), 2–5-fold increase (yellow), 5–10-fold increase (orange), 10–50-fold increase (dark red) and >50-fold increase (black bars). The amount of –fold increase values for each cytokine/chemokine is given as a percentage in the total amount of donors.

For potential microbicidal applications, it is important that LabyA1 has no stimulatory effects on the HIV target cells. Therefore, we incubated freshly isolated PBMCs for 3 days with 9.6 µM of LabyA1 or 0.016 µM of PHA and investigated the expression of the early activation marker CD69 and late activation marker CD25. In untreated conditions, 10.7±3.2% (mean ± SEM) of the cells were CD4^+^CD25^+^ and 1.4±0.8% were CD4^+^CD69^+^ (Fig. 7A). Treatment of the cells with 9.6 µM of LabyA1 had no significant effects on the percentage of activated CD4^+^CD25^+^ and CD4^+^CD69^+^ cells. Treatment of PBMCs with the mitogenic lectin PHA significantly increased the percentage of CD4^+^CD25^+^ and CD4^+^CD69^+^ cells to 37.2±6.6% and 30.9±5.5%, respectively (Fig. 7A).

Activation of CD4^+^ T cells can result in a higher susceptibility for infection with HIV-1. So next, we investigated whether pre-treatment of lymphocytes with LabyA1 has an effect on HIV-1 infectivity. PBMCs were incubated for 24 h with 9.6 and 1.9 µM LabyA1 and 0.078 and 0.016 µM PHA. The cells were subsequently washed and infected with HIV-1 BaL in the absence of compounds. After 7 days, viral replication was measured using HIV-1 p24 Ag ELISA. In the absence of compound, the p24 HIV-1 Ag production was 12.69±4.83 ng/ml (mean ± SEM). Pre-treatment of the cells with 9.6 and 1.9 µM LabyA1 had no significant effect (*p*>0.05) on the degree of infectivity with the HIV-1 R5 strain BaL, with p24-values of 15.37±3.75 and 12.26±4.61 ng/ml, respectively (Fig. 7B). In contrast, a dramatic increase in virus production was observed when the cells were pre-treated with PHA. The viral p24-values increased significantly to 169.54±35.22 ng/ml and 125.08±37.81 ng/ml for 0.078 µM and 0.016 µM PHA (*p*<0.05), respectively. Thus, importantly pre-treatment of PBMCs with LabyA1 did not activate nor influence their viral susceptibility.

Stimulation of PBMCs can result in the induction of cytokines and chemokines. PBMCs were cultured for 24h with LabyA1 or PHA and in the supernatant the concentrations of IL-1α, IL-1ra, IL-2, IL-4, IL-5, IL-6, IL-7, IL-8, IL-9, IL-10, IL-12, IL-13, IL-15, IL-17, eotaxin, FGF, G-CSF, GM-CSF, IFN-γ, IP-10, MCP-1, MIP-1α, MIP-1β, PDGF, RANTES, TNF-α and VEGF were determined. An overview of the degree of drug-induced cytokines/chemokines production is shown in Fig. 7C. The concentration of each cytokine/chemokine was compared with that of the untreated controls and calculated as the fold increase values, which were divided over 5 ranking groups (1, 2–5, 5–10, 10–50, >50-fold increase) indicated by a specific color (Fig. 7C). The cytokine/chemokine response of LabyA1-treated PBMCs was much weaker, if any, compared to the mitogenic lectin PHA.

### Effect of LabyA1 on the Vaginal Epithelial Cells and the Lactobacillus Flora

**Figure 8:**
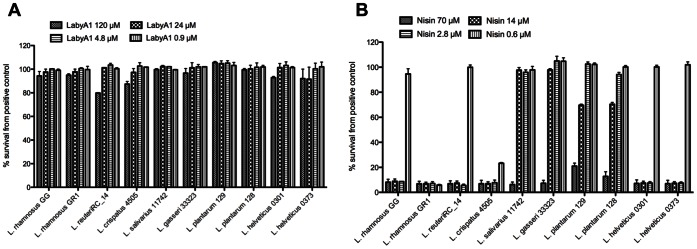
Effect of LabyA1 and nisin on the growth of vaginal lactobacilli sp. Dose-dependent effects of the lantibiotics LabyA1 (A) and nisin (B) on the growth capacity of ten (one gastrointestinal [*L. rhamnosus* GG] and nine vaginal) *Lactobacillus* strains. The results are expressed in percentage survival compared to the growth of each strain in MRS medium without LabyA1 and nisin (positive controls), which were taken as 100%. Mean ± SD of 3 independent experiments is shown.

For potential vaginal microbicidal application it is necessary not to harm the vaginal epithelium or the commensal vaginal *lactobacilli* flora. Therefore various vaginal *Lactobacillus* strains and one gastrointestinal strain (*L. rhamnosus* GG) were exposed to LabyA1 and nisin at different concentrations. At a dose up to 120 µM of LabyA1 no growth inhibitory effects were observed (Fig. 8A). The food preservative nisin, which completely lacked activity against HIV and HSV, killed at the 3 highest concentrations tested (70, 14 and 2.8 µM) many of the vaginal *Lactobacilli* strains (Fig. 8B) [25], [26].

The 50% cytotoxic concentrations (CC_50_) for LabyA1 on the vaginal epithelial cells HEC-1A (endometrium carcinoma) and VK2 (cervix carcinoma) were 34 µM and >48 µM, respectively, as measured by flow cytometry. In addition, we measured also cytotoxicity on various non-epithelial cell lines. The observed CC_50_ values, according to the MTS/PES method were 45 µM in PBMCs, 33 µM in MT-4 cells, 23 µM in C8166 cells, >31 µM in HUT-78 cells, >48 µM in Daudi cells and >48 µM in HEL cells.

### Antiviral Drug Combinations with LabyA1

**Figure 9:**
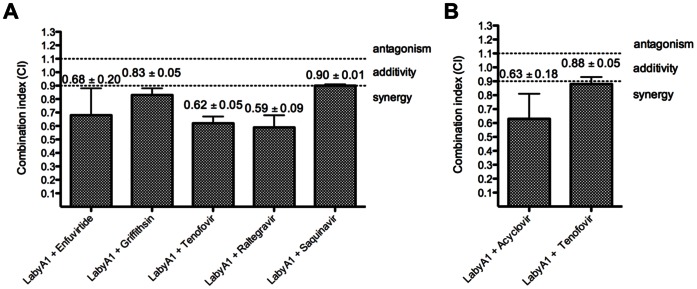
Synergistic activity of LabyA1 with various anti-HIV and anti-HSV compounds. (A) Evaluation of LabyA1 in combination with selected anti-HIV inhibitors of different classes against the R5 HIV-1 clade C strain ETH2220 or clade B strain BaL in PHA-stimulated PBMCs. Mean CIs ± SEM from 2–3 individual donor PBMC experiments are shown. Enfuvirtide and griffithsin are entry inhibitors, tenofovir is a reverse transcriptase inhibitor, raltegravir is an integrase inhibitor and saquinavir is a protease inhibitor. The LabyA1 combination containing tenofovir was evaluated against HIV-1 R5 strain BaL. (B) Evaluation of the LabyA1/acyclovir and LabyA1/tenofovir combinations against HSV-2 induced CPE in HEL cell cultures. Acyclovir and tenofovir are viral DNA polymerase inhibitors. Mean values ± SEM of 2 experiments are shown. The combination indices were calculated at the EC_95_-level whereby CI>1.1 are antagonistic; 0.9<CI<1.1 are additive and CI<0.9 are synergistic. The degree of synergy is further subdivided into: 0.85<CI<0.90: slight synergy (+); 0.7<CI<0.85: moderate synergy (++); 0.3<CI<0.7: synergy (+++) and 0.1<CI<0.3: strong synergy (++++).

Since an effective microbicide will presumably be a combination of at least 2 different compounds, we investigated the effects on HIV replication when LabyA1 is combined with various classes of anti-HIV drugs, and determined the degree of synergism. As shown in Fig. 9A, LabyA1 showed synergism in the dual combinations with the RTI tenofovir, the INI raltegravir and the EI gp41-fusion inhibitor enfuvirtide (T20) and borderline weak synergy to additivity with the PI saquinavir. Moderate synergistic interactions were observed with the potent anti-HIV mannose-specific protein griffithsin (Fig. 9A).

Additionally, we investigated the effects of acyclovir and tenofovir in combination with LabyA1 on HSV-2 replication. As shown in Fig. 9B, slight synergy was observed in combination with tenofovir (CI_95%_, 0.88±0.05), while a better inhibition of viral induced CPE, and thus a lower combination index value (CI_95%_, 0.63±0.18) was obtained with the LabyA1/acyclovir drug combination.

## Discussion

We focused here on the labyrinthopeptins, a novel class of lantibiotics originally isolated from the actinomycete *Actinomadura namibiensis* DSM 6313 [16], [17], [18] and there has been a lot of progress in understanding the biosynthesis of these peptides [16], [19], [20], [21]. Preliminary data showed that the labyrinthopeptins A1 and A2 had activity against herpes simplex virus (HSV) infections *in vitro* [16]. This attracted our interest to investigate whether these peptides also could have anti-HIV activity. As demonstrated here, LabyA1 is the only member of the tested lantibiotics that showed a broad-spectrum anti-HIV activity in various cell-types, irrespective of coreceptor usage (Tables 1 and 2, Fig. 2). It also inhibited the replication of various wild-type and TK-deficient HSV-1 and HSV-2 strains and clinical isolates (Table 3). In fact, the anti-HSV activity of LabyA1 is comparable to the reference compounds acyclovir and cidofovir (Table 3) and importantly, LabyA1 kept its broad-spectrum anti-herpetic activity against acyclovir-resistant strains, as acyclovir (Zovirax®) and valacyclovir (valine ester prodrug of acyclovir; Valtrex®) are the reference compounds for the treatment of HSV-related illnesses (Table 3). The lack of anti-HIV and only moderate anti-HSV activity made LabyA2 a less attractive candidate for further antiviral studies (Fig. 2 and Table 4).

For microbicidal applications, the observed dual antiviral activity of LabyA1 could be of extreme importance, since various studies have shown that HIV transmission and infection is facilitated by other sexually transmitted diseases such as genital HSV-2 [10], [11], [27]. Multiple attempts to find an effective microbicide have failed for many years [28], [29]. However, the South-African CAPRISA 004 trial opened novel perspectives in the field of microbicidal research, whereby it was shown that a 1% tenofovir gel reduced significantly the transmission of HIV by 39% and of HSV-2 by 51% [3]. These data were somewhat surprising since tenofovir was described earlier as a potent anti-HIV and anti-hepatitis B virus (HBV) DNA polymerase inhibitor, with minimal anti-HSV activity *in vitro* [30], [31]. Recently, it has been shown that tenofovir also inhibits the HSV DNA polymerase, although this mechanism of action was only achieved at high drug concentrations [32].

In order to apply LabyA1 as a microbicide against HIV, it is important that it inhibits the various transmission pathways of HIV. The sexual transmission of HIV predominantly occurs by genital secretions (e.g. cervicovaginal fluids, semen), which not only contain cell-free viral particles but also cell-associated virus. Donor infected cells can infect CD4^+^ T cells and here we demonstrated that LabyA1 can inhibit giant cell formation between HIV-infected T cells and uninfected CD4^+^ target T cells in vitro (Fig. 3). In addition, during sexual transmission of HIV, dendritic cells (DCs) that express DC-SIGN can capture HIV particles and transport them to the lymph nodes where the virus is efficiently transmitted to naïve uninfected CD4^+^ T cells [33], [34]. We also demonstrated that LabyA1 could inhibit this cell-mediated HIV transmission process in vitro (Fig. 6). Thus, besides inhibiting cell-free viral infection, LabyA1 is also a potent inhibitor of cell-to-cell- and DC-SIGN-mediated transmission of HIV in vitro. These observations are very important for microbicidal applications against HIV and HSV, as also for HSV it is known to spread through cell-to-cell contacts [12]. To be active in these cellular assays, LabyA1 must interact somewhere between virus attachment to the CD4 receptor and the subsequent viral fusion steps.

To unravel the mechanism of action of LabyA1 against HIV and HSV, time-of-drug addition (TOA) studies were performed, indicating that viral entry is the target area of this peptide (Fig. 4). These data correlate with the results obtained in the HIV cocultivation assay between persistently HIV-infected T cells and uninfected T cells. Based on the fact that LabyA1 does not appear to interact with the CD4 receptor and, in addition, does not inhibit virus binding to CD4^+^ T cells, we can conclude that LabyA1 interferes with HIV entry in a post-CD4 binding event (Fig. 5). Further studies revealed that the drug did not affect the binding of the anti-CXCR4 mAbs clone 12G5 and 2B11 (data not shown) to CXCR4. Also, LabyA1 did not inhibit the chemokine-induced calcium signaling through the CXCR4 or CCR5 receptor nor induce calcium signaling by itself [35]. Instead, surface plasmon resonance (SPR) studies revealed that LabyA1 showed a dose-dependent interaction with R5 and X4 gp120. The binding constants were in the lower µM range, which was comparable with its antiviral activity (Table 5). The lack of cross-resistance with the class of CBAs (e.g. 2G12 mAb) strongly indicates that the N-linked glycans are not a target on gp120 for LabyA1. The exact mechanism of action of LabyA1 against HSV is unknown. Based on the fact that LabyA1 lost its antiviral activity when added 2 h post-HSV-infection indicates that LabyA1 acts as an entry inhibitor possibly by targeting the HSV glycoproteins. This is in agreement with cellular binding experiments (pre-treatment of cells for 1 h and then removing the compound), as in this experimental setup, LabyA1 lost its ability to inhibit HSV-2 replication (data not shown).

An effective microbicide to prevent sexual transmission of HIV will most likely consist of a combination of at least 2 different antiretroviral drugs. Mid 2012, the USA FDA approved the use of tenofovir/emtricitabine (also known as Truvada®) in the PrEP of HIV [36]. LabyA1, tested in combination with clinically approved drugs such as enfuvirtide (T20; gp41 fusion inhibitor), raltegravir (integrase inhibitor) or tenofovir (reverse transcriptase inhibitor), resulted in synergy (Fig. 9A). Also, in combination with the experimental gp120-targeting peptide griffithsin, LabyA1 showed (moderate) synergy. These results were expected regarding the antiviral target of each compound. Why only additive effects were observed in combination with saquinavir is currently not known. Inhibition of HSV-2 infection by combining LabyA1 with acyclovir or tenofovir also resulted in synergy (Fig. 9B). Tenofovir can inhibit HSV-2 replication only at high drug concentrations [32] and this can be an explanation for the weaker degree of synergism observed between LabyA1 and tenofovir (with EC_50s_ >200 µM). Also, the acyclovir/tenofovir combination against HSV-2 showed no synergy (data not shown). A recent study did demonstrate synergistic anti-HSV-2 activity of acyclovir with other classes of antiviral agents such as the helicase-primase inhibitor amenamevir [37]. Griffithsin, the most potent natural occurring peptide with anti-HIV activity in pM range [38], [39], lacks anti-herpes virus activity *in vitro* and was therefore not tested in combination with LabyA1.

An effective microbicide should not stimulate the target CD4^+^ T cells upon exposure to the vaginal environment. In contrast to the mitogenic lectin PHA and the antiviral CV-N lectin, LabyA1 did not activate the cells as demonstrated by the lack of effect on the expression levels of the cellular activation markers CD25 and CD69 (Fig. 7). When PBMCs were pre-incubated with LabyA1 for 24 h and then exposed to R5 HIV-1, no increase in viral replication was observed. Instead, PHA (lacking antiviral activity) and the well-studied anti-HIV lectin CV-N stimulated the CD4^+^ T cells and induced a higher HIV-1 viral replication [40].

It is also very important to investigate the potential harmful effects of a microbicide candidate drug on the vaginal epithelial integrity and the bacterial flora, represented mainly by *Lactobacillus* species [41]. No toxicity on endometrial and cervical epithelial cells was observed. The vaginal *Lactobacilli* play an important role in the defense against various bacterial and viral pathogens such as HIV by reducing the pH to virucidal levels and by the production of hydrogen peroxide [7]. A recent study by Ravel *et al.,* [42], demonstrated that a twice-daily application of vaginal microbicide gels altered the vaginal microbiota, indicating that the evaluation of microbicidal candidates on vaginal microbiota is an important critical endpoint. A concentration of 120 µM of LabyA1 did not have any effects on the growth of a broad variety of vaginal *Lactobacilli*. When nisin, which completely lacks anti-HIV and anti-HSV activity, was evaluated clearly toxic effects on the *Lactobacillus* strains were observed (Fig. 8). As Lipid II serves as a docking molecule for nisin to disrupt the bacterial cell-wall synthesis and to initiate the formation of pores [43], its absence in HIV and HSV could explain the lack of antiviral activity of nisin. Although Aranha *et al.,* have suggested the use of a nisin containing gel in the prophylaxis of sexually transmitted diseases/HIV based on an *in vivo* rabbit model [44], we emphasize that a nisin gel should not be recommended due to its harmful effects on the microbial flora of the vagina. For a probiotic treatment approach, one could investigate the possibility to create LabyA1-expressing *Lactobacillus* strains, as methods for the heterologous expression of LabyA1 have been recently established [45]. A study in macaques showed a reduction of 63% in SHIV transmission after treatment with cyanovirin-N-expressing *Lactobacillus* strains [46].

Finally, an economic access to LabyA1 is important for the desired indication. While strain optimization efforts have not been undertaken so far, we note that even the wild type strain showed a good productivity of 90 mg/l of LabyA1. In addition, a heterologous expression system has been established that enables the production of LabyA1 in *Streptomyces lividans*, a very well characterized host with (unoptimized) titers of 86 mg/l [45]. The expression system also allows the selective knockout of the gene encoding LabyA2. This significantly simplifies the purification of LabyA1, as the chromatographic separation from the closely related LabyA2 component becomes obsolete.

In conclusion, LabyA1 represents a member of a novel class of small-peptide antibiotics that has favorable properties to qualify as a new microbicide drug lead with dual antiviral activity against HIV and HSV infection and transmission.

## Materials and Methods

### Cells and Cell Cultures

The T-lymphocytic CD4^+^ HUT-78, SupT1, C8166 cells were obtained from the American Type Culture Collection (ATCC; Manassas, VA), while the MT-4 cells were a kind gift from Dr. L. Montagnier (formerly at the Pasteur Institute, Paris, France). Persistently HIV-1 X4 IIIB infected HUT-78 cells (HUT-78/IIIB) were generated by infecting HUT-78 cells (5×10^6^ cells) with high amounts of HIV-1 X4 IIIB (∼3.5×10^6^ pg/ml of p24) for 2 h on 37°C. After 3 weeks of cultivation, persistent virus infection was measured using flow cytometry by gp120 staining. The B-lymphocytic Raji.DC-SIGN transfected cells were obtained from Dr. L. Burleigh (Pasteur Institute). Daudi (B lymphoma) cells were obtained from ATCC. All cell lines were cultured in RPMI-1640 medium (Invitrogen, Merelbeke, Belgium) supplemented with 10% fetal calf serum (FCS; Hyclone, Perbio Science, Aalst, Belgium) and 1% l-glutamine (Invitrogen). The human embryonic lung (HEL) fibroblasts, used for the anti-HSV assays, were obtained from the ATCC and cultured in minimum essential medium (MEM; Invitrogen) supplemented with 10% FCS, 1% l-glutamine and 0.3% sodium bicarbonate (Invitrogen). The epithelial cell line HEC-1A was cultivated in DMEM medium supplemented with 10% FCS and 1% HEPES (Invitrogen). The VK2 epithelial cells were cultivated in serum free keratinocyte medium (Invitrogen).

Human astroglioma U87 cells expressing human CD4 (U87.CD4) were a kind gift from Dr. Dan R. Littman (Skirball Institute of Biomolecular Medicine, New York, NY, USA). These cells were transfected with either CCR5 (U87.CD4.CCR5) or CXCR4 (U87.CD4.CXCR4) and cultured in Dulbecco’s modified Eagle’s medium (Invitrogen, Paisley, UK) containing 10% fetal bovine serum (FBS) (BioWhittaker Europe, Verviers, Belgium), 0.01 M HEPES buffer (Invitrogen), 0.2 µg/ml geneticin (G-418 sulfate) (Invitrogen) and 1 µg/ml puromycin (Sigma- Aldrich, St. Louis, MO, USA).

Peripheral blood mononuclear cells (PBMCs) were isolated from buffy coats from healthy blood donors, derived from the blood transfusion center (University Hospitals Leuven), by density gradient centrifugation. The PBMCs were cultured in RPMI-1640 medium containing 10% FCS and 1% l-glutamine. Stimulation was obtained by 2 µg/ml phytohemagglutinin (PHA) for 3 days on 37°C before their further use in anti-HIV assays.

Monocyte-derived macrophages (MDM) were prepared as follows: freshly isolated PBMCs (2×10^6^ cells/ml) were seeded in a 48-well plate (Costar 3548) in 1 ml RPMI-1640 medium with 10% FCS and incubated for 1 week at 37°C. Afterwards, the non-adherent cells were gently mixed and removed from the adherent cell layer. The cells were carefully washed and this washing step was repeated after 6 days of incubation at 37°C. The adherent cells were then infected with HIV-1 R5 BaL.

### Viruses

The HIV-1 strains NL4.3, HIV-1IIIB (both X4) and HIV-1 BaL (R5) were obtained from the AIDS Research and Reference Reagent Program (Division of AIDS, NIAID, NIH). The dual-tropic (R5/X4) HIV-1 strain HE was originally isolated in the Rega Institute from a Belgian AIDS patient and later on cultured in various CD4^+^ T cell lines [47]. Various HIV-1 clinical isolates, representative for different clades, were kindly provided by Dr. J. L. Lathey (then at BBI Biotech Research Laboratories, Gaithersburg, MD) and only passaged in PHA-activated PBMCs. Their co-receptor usage was determined in house using the U87.CD4.CXCR4 and U87.CD4.CCR5-transfected cells. The HIV-1 X4 isolate CI#17 was obtained through the collaboration of the clinical AMD3100 trial via Dr. G. Bridger (at that time AnorMed, Langley, Canada). HIV-2 ROD (R5/X4) was obtained from the Medical Research Council (MRC, London, UK). The *in vitro* generated HIV-1 IIIB or NL4.3 strains resistant to 2G12 mAb, AMD3100, enfuvirtide (T20), raltegravir (RAL), azidothymidinie (AZT) and feglymycin (FGM) were characterized in earlier publications [48], [49], [50], [51].

The HSV-1 strain KOS and HSV-2 strain G were used as reference herpesviruses. Several HSV-1 wild-type (RV-174, RV-175, C559142), HSV-1 thymidine kinase-deficient (TK^−^) (RV-294, RV-179, RV-117), HSV-2 wild-type (RV-124, RV-24) and HSV-2 TK^−^ (RV-129, BA19026589) clinical isolates derived from virus-infected individuals in Belgium were used. More information of the TK^−^ or acyclovir resistant strains can be found in reference [32]. They were obtained as part of a translational research program granted by the Belgian Ministry of Health as part of the National Cancer Plan for the diagnosis of drug resistance in herpesviruses. All viruses were obtained and used as approved according to the rules of Belgian equivalent of IRB (Departement Leefmilieu, Natuur en Energie, protocol SBB 219 2011/0011, and the Biosafety Committee at the KU Leuven).

### Test Agents

Labyrinthopeptins were isolated and purified as described earlier [16]. In brief, LabyA1 was purified by extraction, chromatography and preparative HPLC as a final purification step. The quality of the peptide was checked by UV and NMR spectroscopy and a purity of >99% was obtained. The lantibiotic peptide nisin from *Lactococcus lactis* (MW: 3.5 kDa) was ordered from Sigma-Aldrich (Bornem, Belgium). Griffithsin (GRFT; MW: 25.4 kDa) was a kind gift of Dr. K.E. Palmer (University of Louisville, KY). Human sCD4 was obtained from ImmunoDiagnostics Inc. (Woburn, MA). AMD3100 (MW: 830 g/mol) was a gift from Dr. G. Bridger (at that time AnorMed). Enfuvirtide (T20; MW: 4492 Da) was a kind gift from Dr. E. Van Wijngaerden (UZ Leuven, Belgium). Raltegravir (MW: 482.51 g/mol) was obtained from Tibotec (Mechelen, Belgium). The polyanionic compound dextran sulfate (MW: 8000 g/mol) and the mitogenic lectin phytohemagglutinin (PHA; MW: 128 kDa) were ordered from Sigma-Aldrich (Bornem, Belgium). Tenofovir (MW: 287.21 g/mol) and cidofovir (MW: 315.22 g/mol) were a gift from Gilead Sciences (Foster City, CA). Acyclovir (MW: 225 g/mol) was obtained from GlaxoSmithKline (Brentford, UK) and nevirapine (MW: 266.3 g/mol) was ordered from Boehringer Ingelheim GmbH (Germany).

### Anti-HIV Assays

The antiviral assays in MT-4 cells and PBMCs have been described in detail earlier [52]. Briefly, MT-4 (50 µl, 1×10^6^ cells/ml) were pre-incubated with the compounds (100 µl) for 30 min at 37°C in a 96-well plate. Next, the cell-line adapted HIV strains (NL4.3, IIIB, HE and ROD) were added according to the TCID_50_ (50% tissue culture infectious dose) of the viral stock. After 5 days, cytopathic effect (CPE) was scored microscopically and EC_50_s were calculated using the MTS/PES method [52].

Freshly isolated PBMCs were stimulated with 2 µg/ml PHA for 3 days at 37°C. Then, 5×10^5^ PHA-stimulated PBMCs/ml (200 µl) were seeded in a 48-well plate and pre-incubated for 30 min with 250 µl of test products in the presence of 2 ng/ml IL-2 (Roche Applied Science, Vilvoorde, Belgium) and then (50 µl) 500 pg/well of p24 Ag of virus was added. At days 3 and 6 post viral infection, 2 ng/ml of IL-2 was added. Finally, 10 days post-infection supernatant was collected for p24 HIV-1 (Perkin Elmer, Zaventem, Belgium) or p27 HIV-2 Ag ELISA (INNOTEST, Innogenetics, Temse, Belgium) according to manufacturer’s guidelines.

MDM were seeded in a 48-well plate in 1 ml medium. After removal of 800 µl of cell culture medium (RPMI-1640 supplemented with 10% FCS and 1% l-glutamine), 250 µl of test agent was added. Each concentration was tested in triplicate. After an incubation of 30 minutes at 37°C, 1000 pg/well of p24 Ag of HIV-1 R5 BaL was added. Three weeks post-infection, supernatant was collected and viral replication evaluated by p24 HIV-1 Ag ELISA.

### Giant Cell Cocultivation Assays

The cocultivation experiments were performed as described previously [53]. In brief, LabyA1 was diluted in cell culture medium and 100 µl was added in 96-well plate (BD, Falcon) along with the SupT1 T cells (1×10^5^ cells/50 µl). The same amount of persistently HIV-infected HUT-78/IIIB cells were seeded and incubated at 37°C for 24 h. The next day, giant cell formation was scored microscopically and in addition the depletion of the CD4^+^ SupT1 cells was measured by flow cytometry [53].

### Cytotoxicity Assays

The vaginal epithelial cell lines HEC-1A and VK2 (500 µl; at a density of 1×10^6^ cells/ml) were seeded in a 24-well plate and incubated for 3 days with various concentrations of LabyA1 (500 µl). Cell cytotoxicity was determined microscopically and by flow cytometry (FACSCalibur, BD Biosciences, Erembodegem, Belgium).

Cytotoxicity in PBMCs (3 days), MT-4 (5 days), HUT-78 (1 day), C8166-(5 days), HEL (3 days) and Daudi cells (3 days) was measured using the MTS/PES method [52]. The duration of the assays is given between brackets.

### Anti-HSV Assays

The antiviral assays are based on the inhibition of virus-induced cytopathicity in human embryonic lung (HEL) fibroblasts. Confluent cell cultures in 96-well plates were inoculated with 100 TCID_50_ of virus (1 TCID_50_ being the viral dose to infect 50% of the cell culture) and simultaneously with infection, the cell cultures were incubated in various concentrations of LabyA1, LabyA2, nisin or with the acyclic nucleoside analogues cidofovir and acyclovir as reference compounds for 3 days at 37°C. Viral cytopathicity was measured as soon it reached completion in the control (untreated) virus-infected cell cultures. Anti-HSV activity is expressed as the EC_50_ or compound concentration required to reduce virus-induced cytopathicity by 50%.

### Time-of-drug-addition (TOA) Studies

The time-of-drug-addition (TOA) experiments were performed as described [54]. In brief, 1×10^6^ MT-4 cells/ml were infected with HIV-1 X4 IIIB at a multiplicity of infection (MOI) of 0.5. The compounds were added at different time points in a range from 0 to 26 h post-infection. After 31 h, HIV-1 replication was detected by p24 HIV-1 Ag ELISA as described above. The reference compounds were added at 100 times their EC_50_-values, as obtained in the MT-4 cell antiviral assay.

TOA experiments for HSV-2 were performed identically as the viral replication assays, but each compound separately (LabyA1 or ACV) was added together with the virus (0 h) or after 2 h post-infection. The reference compound was added at least 100 times its EC_50_-value, as obtained in the HEL cell line (Table 3).

### Evaluation of Combined Anti-HIV Products

The method for synergy analysis was described previously [52], [55]. Briefly, first the EC_50_s of LabyA1, tenofovir, saquinavir, raltegravir, enfuvirtide and griffithsin alone were evaluated in PBMCs against R5 HIV-1 ETH2220 (subtype C) or BaL (subtype B). Next, the following LabyA1 combinations (LabyA1/saquinavir, LabyA1/tenofovir, LabyA1/enfuvirtide, LabyA1/raltegravir and LabyA1/griffithsin) were tested against R5 HIV-1 replication. Ten days post-infection, viral replication was measured by p24 HIV-1 Ag ELISA and the combination indices (CIs) were calculated using the CalcuSyn software (Biosoft, Cambridge, UK) based on the median effect principle of Chou and Talalay [56]. For a detailed description of combination studies and synergy calculation, see reference [57].

### Evaluation of Combined Anti-HSV Products

The EC_50_s of LabyA1, acyclovir and tenofovir alone were determined in HEL cell line against HSV-2 strain G as described above. Afterwards, LabyA1 was first combined with acyclovir and then with tenofovir. Viral-induced CPE was scored after 3 days post-infection. The CIs were calculated again by using the CalcuSyn program.

### HIV Binding Assays

The virus binding studies were performed as described previously [51]. Briefly, 200 µl of LabyA1 (9.6 µM), sCD4 (10 µg/ml) and AMD3100 (12 µM) were inserted in a 15 ml polypropylene tube. Subsequently, 200 µl CD4^+^ SupT1 cells (2.5×10^6^ cells/ml) and 100 µl of high amounts of HIV-1 X4 NL4.3 were added (∼2.8×10^6^ pg p24/ml) and incubated for 2 h on room temperature. After washing, virus binding was measured using 500 ng/ml 9205 anti-gp120 mAb (NEA-902, NEN, Boston, MA) and a 1/100 diluted secondary goat-anti-mouse-PE labeled (GaM-PE, Invitrogen) antibody. As a control for aspecific background staining, cells were stained with GaM-PE only. After fixation, the virus binding was measured and analyzed by flow cytometry (FACSCalibur, BD Biosciences) and Cell Quest software (BD Biosciences). Virus binding is expressed in mean fluorescence intensity (MFI) values. Inhibition percentage was calculated after subtracting the background MFI value.

### HIV-1/DC-SIGN-mediated Transmission Assay to Uninfected CD4^+^ T cells

Raji.DC-SIGN^+^ cells (200 µl; 2.5×10^6^ cells/ml) were exposed to high amounts of HIV-1 HE (100 µl; p24, ∼3×10^6^ pg/ml) for 1 h at 37°C. Unbound virus from the Raji.DC-SIGN^+^ cells was removed by washing twice with cell culture medium. In the meantime, 100 µl of various concentrations of LabyA1 were added in a 96-well plate and incubated for 1 h with the C8166 target T cells (50 µl; 2×10^6^ cells/ml). The same amount of virus exposed Raji.DC-SIGN^+^ cells (Raji.DC-SIGN/HE) were mixed with the antiviral drug exposed C8166 target T cells. After 24 h, giant cell formation was scored microscopically and viral replication was determined by HIV-1 p24 Ag ELISA.

### Surface Plasmon Resonance (SPR) Analysis

Recombinant gp120 proteins from X4 HIV-1 IIIB strain (ImmunoDiagnostics Inc., Woburn, MA) and from R5 HIV-1 strains ADA and YU2 (ImmunoDiagnostics Inc.) were covalently immobilized on a CM5 sensor chip in 10 mM sodium acetate, pH 4.0, using standard amine coupling chemistry. The chip densities were 8200 resonance units (RUs), 10760 RUs and 9626 RUs, respectively. A reference flow cell was used as a control for non-specific binding and refractive index changes. All interaction studies were performed at 25°C on a Biacore T200 instrument (GE Healthcare, Uppsala, Sweden). The compounds LabyA1 and nisin were serially diluted in HBS-P (10 mM HEPES, 150 mM NaCl and 0,05% surfactant P20; pH 7.4) supplemented with 5% dimethyl sulfoxide (DMSO, Merck), and 10 mM CaCl_2_ covering a concentration range between 7.8 and 31.3 µM, by using two-fold dilution steps. Samples (in duplicate) were injected for 2 minutes at a flow rate of 45 µl/min and the dissociation was followed for 4 minutes. Several buffer blanks were used for double referencing. The CM5 sensor chip surface was regenerated with a single injection of 50 mM NaOH. A DMSO concentration series was included to eliminate the contribution of DMSO to the measured response. The studied interaction resulted in specific binding signals. The experimental data were fit using the 1∶1 binding model Biacore T200 Evaluation software 1.0 to determine the binding kinetics.

### Flow Cytometry Analyses

To determine the interaction of LabyA1 with CD4, SupT1 cells (100 µl; 3×10^6^ cells/ml) were incubated for 20 min at 4°C with 9.6 µM, 1.9 µM or 0 µM LabyA1. After extensive washing with PBS/FCS2% (PBS supplemented with 2% FCS), anti-CD4-PE conjugated mAbs RPA-T4 (eBioscience, Vienna, Austria), MT441 (Ancell, Enzo Life sciences) and OKT-4 (eBioscience) were added for 30 min at 4°C. For aspecific background staining, cells were incubated with SimulTest™ Control (IgGγ1-FITC/IgGγ2a-PE) (BD Biosciences). After washing, and fixation with 1% formaldehyde solution, samples were analyzed using the FACSCalibur and CellQuest software (BD Biosciences). The same protocol was applied for anti-CXCR4 evaluation using the fluorochrome conjugated mAbs 12G5-PE and 2B11-FITC (both BD Biosciences).

The depletion of the target CD4^+^ SupT1 T cells in the cocultivation assays was measured using PE-conjugated anti-CD28 (BD Biosciences) [53]. The cells were incubated for 30 minutes at room temperature with anti-CD28-PE. After several washing steps, the cells were fixed with a 1% paraformaldehyde solution and analyzed by flow cytometry.

The effects of 9.6 µM LabyA1 and 0.016 µM PHA on the expression levels of the cellular activation markers CD25 and CD69 on PBMCs was measured after 3 days of incubation at 37°C. After washing with PBS/FCS2%, cells were incubated with anti-CD4 conjugated with PerCP (BD Biosciences) and co-stained with the PE-conjugated mAbs anti-CD25 or anti-CD69 (both BD Biosciences) for 30 min at 4°C. For aspecific background staining, cells were incubated with SimulTest™ Control (IgGγ1-FITC/IgGγ2a-PE). After washing, and fixation with 1% formaldehyde solution, samples were analyzed using the FACSCalibur and CellQuest software (BD Biosciences).

### Measurement of Intracellular Calcium Mobilization

Calcium (Ca^2+^) mobilization assays were performed by the use of a fluorometric imaging plate reader (FLIPR) (Molecular Devices, Sunnyvale, CA) as described previously [35]. Briefly, U87.CD4.CCR5 or U87.CD4.CXCR4 cells were digested by trypsin and seeded in gelatine-coated black-wall 96-well microplates at 2×10^4^ cells per well. The next day, the cells were loaded with the fluorescent calcium indicator Fluo-3 acetoxymethyl ester (Molecular Probes, Leiden, The Netherlands) at 4 µM for 45 min at 37°C. Cells were washed 3 times in assay buffer (Hanks’ balanced salt solution with 20 mM HEPES buffer and 0.2% bovine serum albumin, pH 7.4) and incubated for 10 min with Laby A1. The intracellular calcium mobilization induced by LD78β in U87.CD4.CCR5 cells or by SDF-1 in U87.CD4.CXCR4 cells was then measured at 37°C by monitoring the fluorescence as a function of time simultaneously in all the wells. In addition, potential intracellular calcium mobilization induced by Laby A1 was also investigated.

### Effects of LabyA1 on the Susceptibility of PBMCs for HIV-1 Infection

Freshly isolated PBMCs were cultured for 24 h in the presence of various concentrations of LabyA1 and phytohemagglutinin (PHA). The next day, cells were collected, extensively washed in culture medium, resuspended (5×10^5^ cells/450 µl) and seeded in a 48-well plate. Immediately afterwards, 50 µl of HIV-1 R5 strain BaL (500 pg/well) was added and cells were cultures for 7 days. Supernatant was collected 7 days post virus infection and viral replication was determined by p24 HIV-1 Ag ELISA.

### Bio-Plex Cytokine/Chemokine Detection Assay

Freshly isolated PBMCs from 4 individual donors were incubated for 24 h with 9.6 µM LabyA1 or 0.078 µM PHA. The cytokine/chemokine concentration in the cell culture supernatant was determined by the Bio-Plex 200 System (Bio-Rad, Hercules, CA) using the Bio-Plex Human Cytokine 27-Plex assay as described earlier [40].

### Vaginal Lactobacillus Growth Assay

The capacity of different *Lactobacillus* strains (*L. rhamnosus* GG, *L. rhamnosus* GR1, *L. reuteri* RC_14, *L. salivarius* 11742, *L. gasseri* 33323 [all obtained from the ATCC], *L. crispatus* 4505 [obtained from the NCIMB, Aberdeen, Scotland], *L. plantarum* 129, *L. plantarum* 128, *L. helveticus* 0301 and *L. helveticus* 0373 [a gift from Prof. Dr. Mario Vaneechoutte, Laboratory for Bacteriology Research, Ghent University, Belgium]) to grow in the presence of various concentrations of LabyA1 and nisin was determined. *Lactobacillus* strains grown overnight to stationary phase (OD_600 nm_ ∼1.8–2.0) were used as inocula for the assay. The assay was performed in 96-well microtiter plates. Sterile wells were filled with 200 µl MRS (de Man – Rogosa – Sharpe) medium (BD Biosciences). About 0.5×10^7^ colony forming units or CFUs (1/100 of an overnight culture) were added and then 5-fold dilution series of different concentrations of the used compounds were administered. The plates were incubated without shaking for 24 h at 37°C. The OD_600 nm_ was measured after 24 h and as a negative control, each strain was grown in medium without compound (Petrova *et al*., in preparation).

## References

[1] Available: http://www.unaids.org/en/media/unaids/contentassets/documents/epidemiology/2012/gr2012/20121120_UNAIDS_Global_Report_2012_en.pdf.Accessed 2012 Dec 14.

[2] Shattock RJ, Rosenberg Z (2012) Microbicides: topical prevention against HIV. Cold Spring Harb Perspect Med 4: a007385. 10.1101/cshperspect.a007385 PMC3281595 22355798

[3] Abdool Karim Q, Abdool Karim SS, Frohlich JA, Grobler AC, Baxter C, et al (2010) Effectiveness and safety of tenofovir gel, an antiretroviral microbicide for the prevention of HIV infection in women. Science 329: 1168–1174. 20643915 10.1126/science.1193748 PMC3001187

[4] Celum CL (2011) HIV preexposure prophylaxis: new data and potential use. Top Antivir Med 19: 181–185. 22298887 PMC6148898

[5] Nel AM, Coplan P, Smythe SC, McCord K, Mitchnick M, et al (2010) Pharmacokinetic assessment of dapivirine vaginal microbicide gel in healthy, HIV-negative women. AIDS Res Hum Retroviruses 26: 1181–1190. 20854207 10.1089/aid.2009.0227

[6] Romano J, Variano B, Coplan P, Van Roey J, Douville K, et al (2010) Safety and availability of dapivirine (TMC120) delivered from an intravaginal ring. AIDS Res Hum Retroviruses 25: 483–488. 10.1089/aid.2008.0184 19388819

[7] Shattock RJ, Moore JP (2003) Inhibiting sexual transmission of HIV-1 infection. Nat Rev Microbiol 1: 25–34. 15040177 10.1038/nrmicro729

[8] Ariën KK, Jespers V, Vanham G (2011) HIV sexual transmission and microbicides. Rev Med Virol 21: 110–133. 21412935 10.1002/rmv.684

[9] Tilton JC, Doms RW (2010) Entry inhibitors in the treatment of HIV-1 infection. Antiviral Res 85: 91–100. 19683546 10.1016/j.antiviral.2009.07.022

[10] Corey L (2007) Synergistic copathogens – HIV-1 and HSV-2. N Engl J Med 356: 854–856. 17314346 10.1056/NEJMe068302

[11] Thurman AR, Doncel GF (2012) Herpes simplex virus and HIV: genital infection synergy and novel approaches to dual prevention. Int J STD AIDS 23: 613–619. 23033511 10.1258/ijsa.2012.011356

[12] Karasneh GA, Shukla D (2011) Herpes simplex virus infects most cell types in vitro: clues to its success. Virol J 8: 841. 10.1186/1743-422X-8-481 PMC3223518 22029482

[13] Connolly SA, Jackson JO, Jardetzky TS, Longnecker R (2011) Fusing structure and function: a structural view of the herpesvirus entry machinery. Nat Rev Microbiol 9: 369–381. 21478902 10.1038/nrmicro2548 PMC3242325

[14] Willey JM, van der Donk WA (2007) Lantibiotics: peptides of diverse structure and function. Annu Rev Microbiol 61: 477–501. 17506681 10.1146/annurev.micro.61.080706.093501

[15] Cotter PD, Hill C, Ross RP (2005) Bacteriocins: developing innate immunity for food. Nat Rev Microbiol 3: 777–788. 16205711 10.1038/nrmicro1273

[16] Meindl K, Schmiederer T, Schneider K, Reicke A, Butz D, et al (2010) Labyrinthopeptins: a new class of carbacyclic lantibiotics. Angew Chem Int Ed Engl 49: 1151–1154. 20082397 10.1002/anie.200905773

[17] Seibert G, Vértesy L, Wink J, Winkler I, Süssmuth R, et al.. (2008) WO2008/040469.

[18] Wink J, Kroppenstedt RM, Seibert G, Stackebrandt E (2003) Actinomadura namibiensis sp. nov. Int J Syst Evol Microbiol 53: 721–724. 12807192 10.1099/ijs.0.02286-0

[19] Müller WM, Schmiederer T, Ensle P, Süssmuth RD (2010) In vitro biosynthesis of the prepeptide of type-III lantibiotic labyrinthopeptin A2 including formation of a C-C bond as a post-translational modification. Angew Chem Int Ed Engl 49: 2436–2440. 20191635 10.1002/anie.200905909

[20] Müller WM, Ensle P, Krawczyk B, Süssmuth RD (2011) Leader peptide-directed processing of Labyrinthopeptin A2 precursor peptide by the modifying enzyme LabKC. Biochemistry 50: 8362–8373. 21905643 10.1021/bi200526q

[21] Krawczyk B, Ensle P, Müller W, Süssmuth RD (2012) Deuterium labeled peptides give insights into the directionality of class III lantibiotic synthetase LabKC. JACS 134: 9922–9925. 10.1021/ja3040224 22687055

[22] Skinner MA, Ting R, Langlois AJ, Weinhold KJ, Lyerly HK, et al (1988) Characteristics of a neutralizing monoclonal antibody to the HIV envelope glycoprotein. AIDS Res Hum Retroviruses 4: 187–197. 2456088 10.1089/aid.1988.4.187

[23] Schols D, Struyf S, Van Damme J, Esté JA, Henson G, et al (1997) Inhibition of T-tropic HIV strains by selective antagonization of the chemokine receptor CXCR4. J Exp Med 186: 1383–1388. 9334378 10.1084/jem.186.8.1383 PMC2199084

[24] Hoorelbeke B, Huskens D, Férir G, François KO, Takahashi A, et al (2010) Actinohivin, a broadly neutralizing prokaryotic lectin, inhibits HIV-1 infection by specifically targeting high-mannose-type glycans on the gp120 envelope. Antimicrob Agents Chemother 54: 3287–3301. 20498311 10.1128/AAC.00254-10 PMC2916299

[25] Choi MH, Park YH (2000) Selective control of lactobacilli in kimchi with nisin. Lett Appl Microbiol 30: 173–177. 10747245 10.1046/j.1472-765x.2000.00719.x

[26] Chun W, Hancock RE (2000) Action of lysozyme and nisin mixtures against lactic acid bacteria. Int J Food Microbiol 60: 25–32. 11014519 10.1016/s0168-1605(00)00330-5

[27] Blower S, Ma L (2004) Calculating the contribution of herpes simplex virus type 2 epidemics to increasing HIV incidence: treatment implications. Clin Infect Dis 39 (S5): S240–S247. 10.1086/422361 15494895

[28] Abdool Karim SS, Richardson BA, Ramjee G, Hoffman IF, Chirenje ZM, et al (2011) Safety and effectiveness of BufferGel and 0.5% PRO2000 gel for the prevention of HIV infection in women. AIDS 25: 957–966. 21330907 10.1097/QAD.0b013e32834541d9 PMC3083640

[29] Van Damme L, Ramjee G, Alary M, Vuylsteke B, Chandeying V, et al (2002) Effectiveness of COL-1492, a nonoxynol-9 vaginal gel, on HIV-1 transmission in female sex workers: a randomised controlled trial. Lancet 360: 971–977. 12383665 10.1016/s0140-6736(02)11079-8

[30] Balzarini J, Holý A, Jindrich J, Naesens L, Snoeck R, et al (1993) Differential antiherpesvirus and antiretrovirus effects of the (S) and (R) enantiomers of acyclic nucleoside phosphonates: potent and selective in vitro and in vivo antiretrovirus activities of (R)-9-(2-phosphonomethoxypropyl)-2,6-diaminopurine. Antimicrob Agents Chemother 37: 332–338. 8452366 10.1128/aac.37.2.332 PMC187663

[31] Ying C, De Clercq E, Neyts J (2000) Lamivudine, adefovir and tenofovir exhibit long-lasting anti-hepatitis B virus activity in cell culture. J Viral Hepat 7: 79–83. 10718947 10.1046/j.1365-2893.2000.00192.x

[32] Andrei G, Lisco A, Vanpouille C, Introini A, Balestra E, et al (2011) Topical tenofovir, a microbicide effective against HIV, inhibits herpes simplex virus-2 replication. Cell Host Microbe 10: 379–389. 22018238 10.1016/j.chom.2011.08.015 PMC3201796

[33] Geijtenbeek TB, Van Kooyk Y (2003) DC-SIGN: a novel HIV receptor on DCs that mediates HIV-1 transmission. Curr Top Microbiol Immunol 276: 31–54. 12797442 10.1007/978-3-662-06508-2_2

[34] Hladik F, McElrath MJ (2008) Setting the stage: host invasion by HIV. Nat Rev Immunol 8: 447–457. 18469831 10.1038/nri2302 PMC2587276

[35] Princen K, Hatse S, Vermeire K, De Clercq E, Schols D (2004) Evaluation of SDF-1/CXCR4-induced Ca2+ signaling by fluorometric imaging plate reader (FLIPR) and flow cytometry. Cytometry A 51: 35–45. 10.1002/cyto.a.10008 12500303

[36] Centers for Disease Control and Prevention (CDC) (2012) Interim Guidance for Clinicians Considering the Use of Preexposure Prophylaxis for the Prevention of HIV Infection in Heterosexually Active Adults. MMWR Morb Mortal Wkly Rep 61: 586–589. 22874836

[37] Chono K, Katsumata K, Suzuki H, Shiraki K (2013) Synergistic activity of amenamevir (ASP2151) with nucleoside analogs against herpes simplex virus types 1 and 2 and varicella-zoster virus. Antiviral Res. 97: 154–160. 10.1016/j.antiviral.2012.12.006 23261844

[38] Mori T, O’keefe BR, Sowder RC 2nd, Bringans S, Gardella R, et al (2005) Isolation and characterization of griffithsin, a novel HIV-inactivating protein, from the red alga Griffithsia sp. J Biol Chem 280: 9345–9353. 15613479 10.1074/jbc.M411122200

[39] Emau P, Tian B, O’keefe BR, Mori T, McMahon JB, et al (2007) Griffithsin, a potent HIV entry inhibitor, is an excellent candidate for anti-HIV microbicide. J Med Primatol 36: 244–253. 17669213 10.1111/j.1600-0684.2007.00242.x

[40] Huskens D, Vermeire K, Vandemeulebroucke E, Balzarini J, Schols D (2008) Safety concerns for the potential use of cyanovirin-N as microbicidal anti-HIV agent. Int J Biochem Cell Biol 40: 2802–2814. 18598778 10.1016/j.biocel.2008.05.023

[41] Ravel J, Gajer P, Abdo Z, Schneider GM, Koenig SS, et al (2011) Vaginal microbiome of reproductive-age women. Proc Natl Acad Sci U S A 108 Suppl 14680–4687. 20534435 10.1073/pnas.1002611107 PMC3063603

[42] Ravel J, Gajer P, Li F, Mauck CK, Koenig SS, et al (2012) Twice-daily application of HIV microbicides alter the vaginal microbiota. MBio 3: e00370–12. 23249810 10.1128/mBio.00370-12 PMC3529542

[43] Breukink E, De Kruijff B (2006) Lipid II as target for antibiotics. Nat Rev Drug Discov 5: 321–332. 16531990 10.1038/nrd2004

[44] Aranha CC, Gupta SM, Reddy KV (2008) Assessment of cervicovaginal cytokine levels following exposure to microbicide nisin gel in rabbits. Cytokine 43: 63–70. 18513989 10.1016/j.cyto.2008.04.005

[45] Krawczyk JM, Völler GH, Krawczyk B, Kretz J, Brönstrup M, et al.. (2013) Heterologous expression and engineering studies of labyrinthopeptins, new class III lantibiotics from Actinomadura namibiensis. Chem Biol. In press. 10.1016/j.chembiol.2012.10.023 23352145

[46] Lagenaur LA, Sanders-Beer BE, Brichacek B, Pal R, Liu X, et al (2011) Prevention of vaginal SHIV transmission in macaques by a live recombinant Lactobacillus. Mucosal Immunol 4: 648–657. 21734653 10.1038/mi.2011.30 PMC3433722

[47] Pauwels R, Andries K Desmyter J, Schols D, Kukla MJ, et al (1990) Potent and selective inhibition of HIV-1 replication in vitro by a novel series of TIBO derivatives. Nature 343: 470–474. 1689015 10.1038/343470a0

[48] Huskens D, Van Laethem K, Vermeire K, Balzarini J, Schols D (2007) Resistance of HIV-1 to the broadly HIV-1-neutralizing, anti-carbohydrate antibody 2G12. Virology 360: 294–304. 17123566 10.1016/j.virol.2006.10.027

[49] De Vreese K, Kofler-Mongold V, Leutgeb C, Weber V, Vermeire K, et al (1996) The molecular target of bicyclams, potent inhibitors of human immunodeficiency virus replication. J Virol 70: 689–696. 8551604 10.1128/jvi.70.2.689-696.1996 PMC189868

[50] Goethals O, Vos A, Van Ginderen M, Geluykens P, Smits V, et al (2010) Primary mutations selected in vitro with raltegravir confer large fold changes in susceptibility to first-generation integrase inhibitors, but minor fold changes to inhibitors with second-generation resistance profiles. Virology 402: 338–346. 20421122 10.1016/j.virol.2010.03.034

[51] Férir G, Hänchen A, François KO, Hoorelbeke B, Huskens D, et al (2012) Feglymycin, a unique natural bacterial antibiotic peptide, inhibits HIV entry by targeting the viral envelope protein gp120. Virology 433: 308–319. 22959895 10.1016/j.virol.2012.08.007

[52] Vermeire K, Princen K, Hatse S, De Clercq E, Dey K, et al (2004) CADA, a novel CD4-targeted HIV inhibitor, is synergistic with various anti-HIV drugs in vitro. AIDS 18: 2115–2125. 15577644 10.1097/00002030-200411050-00003

[53] Férir G, Palmer KE, Schols D (2012) Griffithsin, alone and combined with all classes of antiretroviral drugs, potently inhibits HIV cell-cell transmission and destruction of CD4+ T cells. J Antivir Antiretrovir. 4: 103–112.

[54] Daelemans D, Pauwels R, De Clercq E, Pannecouque C (2011) A time-of-drug addition approach to target identification of antiviral compounds. Nat Protoc 6: 925–933. 21637207 10.1038/nprot.2011.330 PMC7086561

[55] Férir G, Palmer KE, Schols D (2011) Synergistic activity profile of griffithsin in combination with tenofovir, maraviroc and enfuvirtide against HIV-1 clade C. Virology. 417: 253–258. 10.1016/j.virol.2011.07.004 21802104

[56] Chou TC, Talalay P (1984) Quantitative analysis of dose-effect relationships: the combined effects of multiple drug or enzyme inhibitors. Adv Enzyme Regul 22: 27–55. 6382953 10.1016/0065-2571(84)90007-4

[57] Chou TC (2006) Theoretical basis, experimental design, and computerized simulation of synergism and antagonism in drug combination studies. Pharmacol Rev. 58: 621–681. 10.1124/pr.58.3.10 16968952

